# The importance of combining open-ended and closed-ended questions when conducting patient satisfaction surveys in hospitals

**DOI:** 10.1016/j.hpopen.2021.100033

**Published:** 2021-01-21

**Authors:** Keren Semyonov-Tal, Noah Lewin-Epstein

**Affiliations:** a Department of Labor Studies, Tel Aviv University, Israel; b Department of Sociology, Tel Aviv University, Israel

**Keywords:** Patient satisfaction, Patient voice, Qualitative & quantitative methods

## Abstract

**Background:**

Using data obtained from the National Satisfaction Survey in General Hospitals, 2014, the present study examines patients’ satisfaction with medical care and hospitalization conditions in the public hospitals in Israel. Using the framework of ‘voice’ expression the study examines the added-value of analyzing verbal responses to gage patient satisfaction.

**Methods:**

The analysis utilizes a series of closed-ended questions to construct indexes of patients’ satisfaction with medical doctors, nursing staff, and hospitalization conditions for a sample of 11,098 patients who were hospitalized in the 25 public hospitals. In addition, a content analysis was applied to the verbal responses (open ended question) to create categories of complaints. Using logistic regression models, we analyzed the social and demographic correlates of high satisfaction, and estimated the relationship between verbal complaints and satisfaction scores.

**Results:**

Analysis of the satisfaction measures shows very high levels of patient satisfaction coupled with low variance. Yet, detailed analysis of responses to an open-ended question reveals considerably more critical assessments of the hospitalization experience.

**Conclusion:**

The findings illustrate the limitations of closed-ended satisfaction items as the sole instrument for assessing the quality of medical care and underscore the value of the use of mixed methods as a more nuanced approach.

## Highlights

• Analysis of patient satisfaction measures shows very high levels of patient satisfaction.
• Analysis also show low variance between patients.
• Open-ended questions reveal considerably more critical assessments of the hospitalization experience.
• Closed-ended satisfaction questions are limited as sole instrument for assessing medical care.
• The findings underscore the value of mixed methods in health studies.

## 1 Introduction

Patients’ evaluation of the hospitalization experience has become an important tool in assessment of quality of medical care [1], [2], [3]. Consequently, health organizations invest much resources in the development of satisfaction surveys with the goal of achieving high level of patients’ satisfaction. Currently, satisfaction surveys are widely used by hospitals across the globe to monitor and evaluate the quality of care [4], [5], [6], [7]. High level of satisfaction with medical care is taken to reflect high quality of medical care and vice versa.

Although satisfaction surveys are widely used, they have well-known limitations [8], [9]. First and foremost, satisfaction scores tend to be positively biased and with little variation [1], [10]. Reliance on satisfaction scores as sole indicators of quality of care can be a cause for concern because survey data by itself may provide only limited valid information. By contrast, information obtained by mixed methods is likely to be richer and more valid [11], [12], [13], [14], [15], [16].

The purpose of this study is to explore patients' perceptions of quality of care provided in public hospitals in Israel. We do so by analyzing patients’ satisfaction, using standard closed-ended questions and by studying patient’s responses to an open-ended question regarding the hospitalization experience. The primary question we address, then, is whether verbal reports of patients add substantively to our understanding of patients’ hospital experience in and above the commonly used quantitative indicators of satisfaction. To the best of our knowledge, such analysis has not yet been conducted on the basis of large population-wide survey and we believe that this line of research provides an important contribution to the development of knowledge in the field.

### 1.1 Satisfaction with medical care

Whereas some researches find value in patients’ subjective assessment of medical treatment [7], [17], [18], [19], [4], [34], others raise doubts about the relationship between satisfaction and quality of care [10], [20], [21]. Patient satisfaction surveys are also used to study other aspects of the system such as availability and length of waiting time for treatment. Even if one accepts the view that patient satisfaction is related to quality of treatment it is important to note that it is only one of several indicators used to evaluate quality of care in the health care system. The doubts are based on the premise that patients lack the necessary medical knowledge to accurately evaluate and fully understand the quality of medical care [20], [22]. According to this view, satisfaction surveys reflect, at best, personal experience of medical treatment and cannot be used as valid indicators of quality of treatment. Patient satisfaction can serve as an important indicator of quality of treatment but is just one of many components. Surveys can be used to collect data on processes and outcomes of care, not just satisfaction. Some of these processes and outcomes are component of quality. Yet, there are other important indicators of quality (e.g. infection rates, duration of surgical operations, mortality) for which surveys are not a good data source and for which clinical/administrative databases are to be preferred.

Taking a broader view, Tucker [23] argues that patient satisfaction research suffers from a lack of an explicitly stated theory and that it explains little of the differences between groups. Others argue that level of satisfaction is influenced by personal characteristics of patients [24], [25] but Tucker and Adams [26] demonstrate that socio-demographic characteristics account for less than 1% of the explained variance in satisfaction. Furthermore, Cleary & McNeil [10] argue that surveys of patient satisfaction commonly reveal very high satisfaction scores coupled with little variation. In order to remedy some of these limitations’ researchers suggest that the use of mixed methods (qualitative alongside quantitative instruments) may offer more fruitful assessment of patients' hospital experiences [11], [12], [13].

### 1.2 Voice, silence and exiting medical treatment

While quantitative measures of satisfaction based on closed-ended items are most common, there are alternatives ways of gaging people's satisfaction. One straightforward way of doing so is by asking patients to express in their own words dis-satisfaction. That is, to reveal ‘their true views’ through an expression of “voice” [27], [28], [29]. Writing about customer satisfaction, in general, Hirschman [28] presents three possibilities for ‘'handling' dissatisfaction with service: the first: ‘voice’ (crying out dissatisfaction), the second ‘silence’ (suppressing dissatisfaction) and the third- ‘exit’ (exiting the relationship due to dissatisfaction) without expressing voice. According to this approach the patient is viewed as a 'service recipient' or a 'consumer' and caregivers and hospital staff are viewed as 'service providers'. Given the opportunity, patients may decide to voice their grievances regarding the quality of care received within the medical system or remain silent. Exit is rarely an option in healthcare situation, thus leaving the patient with two options - to voice their dissatisfaction, or not to do so [30], [31], [32].

Following this line of logic, patient responses to open ended questions are an expression of ‘voice’ [28], [29] whereas a response to a closed ended question is a lower quality expression of voice. The fact that closed ended questions are based on researcher-provided responses may prevent respondents to fully and accurately express their feelings. Following this logic, in the analysis that follows we examine both satisfaction (closed ended questions) and expression of voice (open ended question) in order to advance knowledge in this field of research. We propose that high prevalence of (negative) ‘voice-expression’ reveals high levels of dissatisfaction whereas ‘no expression of voice’ typically represents satisfaction with the quality of care.

### 1.3 The Israeli context

The health care system in Israel, publicly funded by national health insurance, includes 33 general public hospitals scattered throughout the country and an elaborate network of clinics, pharmacies, and preventive medical services. The National Health Law guarantees a defined and equal “basket” of health services for all Israeli residents. Inclusion of all residents under the rule of law insures all patients’ equal accessibility to hospital health care [33]. In this context of, mostly, publicly funded and fairly homogeneous health care system, the study aims to examine satisfaction with hospitalization conditions across population groups in Israel, and the relationship between quantitative indicators of satisfaction and verbal expressions of voice.

## 2 Method

The study uses the, “National Satisfaction Survey in General Hospitals, 2014“, which was collected by the Israeli Ministry of Health that year. The sample consists of 11,098 patients who were hospitalized in the 25 public hospitals (of the 33 general hospitals in Israel). Data were collected within 14 days following patients release from the hospital. Three satisfaction indicators were constructed to capture the hospitalization experience: satisfaction with treatment by physicians, satisfaction with the care provided by nurses, and satisfaction with hospitalization conditions. Each of the three measures of satisfaction was constructed as the average of responses to 4 closed-ended questions, each with a five-category answer scale ranging from 5 (very satisfied) to 1 (not at all satisfied). The Nursing Services Satisfaction Index was constructed as an index of the following questions (Alfa Cronbach = 0.8150):1. From the moment you arrived at the department, to what extent was the department's admissions process effective; 2. During your most recent hospitalization, to what extent did you feel the nurses treated you graciously and respectfully;3. To what extent did the nurses listen to you and addressed your questions and concerns;4. The extent to which the explanations you received during hospitalization were clear and understandable to you; Physician satisfaction were constructed of the following questions (Alfa Cronbach = 0.9030):1. During your most recent hospitalization, to what extent did you feel the doctors treated you kindly and respectfully; 2. To what extent did you feel that you were being treated personally by the doctors?; 3. To what extent did the doctors listen to you and address your questions and concerns?; 4. To what extent did the explanations you received during the hospitalization from the doctors clear and understandable to you?; The Environmental Satisfaction was constructed of the following questions (Alfa Cronbach = 0.7260):1. How satisfied are you with the conditions in your room;2. During the hospitalization to what extent was it quiet during the night;3. How satisfied were you with the food you were served during the hospitalization;4. To what extent were visitors conditions comfortable and appropriate. We did not find a high correlation between the three indices. In addition to closed-ended satisfaction items, respondents were asked to add comments and suggestions in responses to an open-ended question saying: “*Do you have any further comments or suggestions for improvement?*”. We make use of verbal responses to this question in order to interrogate the meaning attached to the closed-ended satisfaction items and to capture gaps between numeric and verbal responses.^1^

The independent variables that are used as predictors of the satisfaction indices are patient's personal socio-demographic characteristics. They include: gender (male = 1), education (academic education = 1), and ethnic origin (Jews = 1). In addition, we include the following health related variables for control purpose: age (in years), medical condition on the eve of hospitalization (chronic illness = 1). Several variables that are related to the conditions of hospitalization are also included in the analysis. These are, whether or not the person was able to choose the hospital (had choice of hospital = 1), and accommodation within the hospital (in a room = 1, in the corridor = 0).

## 3 Results

We begin by examining the distribution of patients’ satisfaction with respect to 3 dimensions of their hospital experience. Table 1 provides the list of satisfaction measures, their definitions, and descriptive statistics. The findings reveal very high mean scores reflecting a strong tendency to report high levels of satisfaction, especially regarding treatment of nurses and doctors. Concomitantly, the standard deviations are small in all three satisfaction scales (nurse care: 4.37 (std 0.72); physician care: 4.30 (std 0.81); and hospitalization conditions: 3.95 (std 0.80), indicating little variability in patients’ responses to the satisfaction items.

**Table 1:** Variable used in the analysis, definitions, and mean (s.d.) or percentage (N =11,098)

Variable	Definition	Mean (sd) or percentage
Satisfaction with treatment by nurses	Average of four answers, according to the scale - the number 5 = very satisfied and the number 1 = not at all satisfied. I assumed that the scale was interval	4.37 (0.72)
Satisfaction with treatment by physicians	Average of four answers, according to the scale - the number 5 = very satisfied and the number 1 = not at all satisfied. I assumed that the scale was interval	4.3 (0.81)
Satisfaction with conditions of hospitalization	Average of four answers, according to the scale - the number 5 = very satisfied and the number 1 = not at all satisfied. I assumed that the scale was interval	3.95 (0.80)

Although all measures show high satisfaction, the interrelationships among the three satisfaction measures are only moderate: Pearson correlation estimates range from 0.62 between satisfaction with physicians and the nursing staff; to 0.44 with respect to satisfaction with physicians and hospitalization conditions. The correlation estimates suggest that subjective evaluation of hospital care is not uniform and that patients are sensitive to the various dimensions of care when responding to the satisfaction questionnaire.

In order to explore the correlates of satisfaction we turn to multivariate modeling of responses to the satisfaction questions. In view of the skewed distribution of the scales we chose not to use linear modeling which focuses on the mean of the distribution and variation around, but rather to explore the likelihood of expressing high satisfaction. To this end, we dichotomized the satisfaction scores contrasting satisfaction with the hospitalization experience (scores 4 and 5 on the response scale) with less favorable responses (scores 1 through 3). We then fitted logistic regression with individual-level sociodemographic characteristics (ethnic origin, gender, and education), health-status, and hospitalization environment as predictors of the likelihood of high vs. low satisfaction separately for nursing care, doctors' treatment, and the hospitalization conditions. The results of the logistic regression analysis are presented in Table 2, model 1. Linear regression predicting satisfaction on 5-point scale produces highly similar results.

**Table 2:** Coefficients of logit regression equations for predicting high vs. low inpatient satisfaction with nurses, doctors and environment.

Variables	Nurse satisfaction		Doctor satisfaction		Environmental satisfaction	
	Model 1	Model 2	Model 1	Model 2	Model 1	Model 2
*Constant*	1.041**	1.411**	1.178**	1.588**	−0.759**	**−**0.435**
*Gender (male = 1)*	0.113**	0.081	0.038	**−**0.001	0.245**	0.211**
*Education (academic =* *1)*	**−**0.159**	**−**0.115*	**−**0.150**	**−**0.102*	**−**0.235**	**−**0.187**
*Ethnicity (Jew = 1)*	**−**0.485**	**−**0.391**	**−**0.776**	**−**0.692**	**−**0.654**	**−**0.569**
*Age*	**−**0.005**	**−**0.007**	0.001	**−**0.001	0.009**	0.007**
*Chronic (chronic = 1)*	**−**0.084	**−**0.054	**−**0.101**	**−**0.070	**−**0.074	**−**0.033
*Accommodation (room = 1)*	0.598**	0.598**	0.358**	0.338**	0.504**	0.474**
*Hospital choice(choice =* *1)*	0.210**	0.186**	0.249**	0.226**	0.172**	0.157**
No complaint	–	–	–	–	–	–
Complaint treatment	–	**−**1.135**	–	**−**1.265**	–	**−**0.650**
Complaint environment	–	**−**0.269**	–	**−**0.250**	–	**−**0.946**
Log likelihood	12386	11948.27	12387	11833.82	14681	14299.61
Cox & Snell R-square	0.022	0.060	0.024	0.072	0.034	0.067
Nagelkereke R-square	0.032	0.088	0.035	0.106	0.045	0.089
*N = 11,198*						

*P < .05, **P < .000.

Although most coefficient estimates are statistically significant, suggesting that satisfaction is related to patient’s social attributes and hospitalization conditions, but less so to respondents’ age and health condition, the models provide rather poor fit. That is, characteristics of patients and the hospital in which they were hospitalized are not very good predictors of satisfaction. This is consistent with previous studies on satisfaction with health care in hospitals which showed that that only a small portion of the variance in satisfaction with health care could be explained [23].

Specifically, the findings do not lend support to the hypotheses that satisfaction (as a measure of quality of care) is lower among less advantaged populations such as less educated persons or the Arab minority (the subordinate ethnic group in Israel) than among more advantaged population group. In fact, the better educated and Jews appear to express lower satisfaction, possibly entering the system with higher expectations. The findings in Table 2 also show that conditions of hospitalization have cross over, albeit small, effects as patients who were accommodated in rooms were more satisfied not only with hospitalization conditions but with treatment by doctors and nurses as well. Similarly, patients who had a choice of hospital are more satisfied overall than patients who did not.

### 3.1 Voicing dissatisfaction

As noted earlier, respondents were offered the opportunity to provide additional comments and concerns in response to an open-ended question: “*Do you have any further comments or suggestions for improvement?*”. Of the 11,098 patients who responded to the survey only 4825 patients provided verbal answers. All verbal responses were coded and were searched for common themes. They were then classified into 9 broad categories according to the nature of the complaint or criticism. The vast majority of patients referred to one issue (made one comment), and few expressed views on 2 or 3 different issues. For this research only the first comment was entered, after considering that the first comment made is usually the most burning issue for the patient. The classification of the verbal comments into categories is presented in Table 3.

**Table 3:** Distribution of voice expression of first comment by categories by type.

Numerical code	Theme	Narratives	Number of cases (%)	Percent of verbal responses
	All cases		*11098 (100.0)*	
**0**	Silence/ no voice	No verbal comment was given	6273 (56.5)	
**1-9**	Expression of voice	Verbal comment was given	4825 (43.5)	100.0
**1**	Positive voice	A verbal comment of positive nature was given	671	13.9
**2**	Complaints / comments regarding physical conditions	Level of cleanliness, adjustment of conditions to medical condition, keeping quiet at night, room / ward density, accommodation conditions of escorts), amount of visitors, visitor behavior, security and theft	1419	29.4
**3**	Complaints on caregiver relationship	Criticism of interpersonal conduct	756	15.7
**4**	Complaints about the nature of food	Review on food quality, cleanliness and food serving	560	11.6
**5**	Complaints on medical staff availability	Comments / Complaints on Personnel Lack / Staff Availability, Therapeutic Bureaucracy, Release Bureaucracy, Medical Care Availability	474	9.8
**6**	Complaints on Emergency Room	Complaint/s/ comments on Emergency Room Care and Management	401	8.3
**7**	Complaints on medical malpractice	Comments / Complaints about Medical Malpractice, Diagnosis or Misuse or Medical Treatment, Unqualified / Professional Medical Care	160	3.3
**8**	Complaints on communication with medical staff	Comments / complaints about the lack of proper communication / lack of knowledge / explanatory quality, both during treatment and in the release phase -	353	7.3
**9**	Complaints on maintaining privacy and respect	Comments / complaints about failure to maintain privacy / physical / personal respect	30	0.6
			4825	100.0

As is evident from Table 3, 56.5% chose not to answer the open-ended question, while 43.5% provide a verbal answer. 13.8% of respondents who answered the open-ended question expressed a positive opinion on the inpatient experience whereas the others expressed a negative opinion concerning at least one aspect of the hospitalization experience. The very small number of positive verbal comments suggests that patients are more likely to make use of the opportunity to offer verbal comments primarily when they were dissatisfied with the treatment or conditions of hospitalization. Of those who expressed an opinion, 41% criticized the hospitalization setting (conditions & food) and the latter (45.1%) criticized the medical treatment. Even if we assume that all those who did not answer the open-ended question held a generally positive attitude, this still leaves over 43% of respondents who were dissatisfied to one extent or another. This seems at odds with the very high levels of satisfaction we observed using the standard quantitative measures.

In order to gain a better understanding of the tendency of certain patients to verbally express their voice we investigate the correlates of expressing negatives views, positive views or no views at all. To do so we estimated a logistic regression model with a binary outcome. Since preliminary analysis revealed no distinction between category of positive comments and the category of no verbal comment, we combined the two for the regression analysis. Hence, the equation provides estimates of the likelihood of expressing dissatisfaction as a function of the associated variables (gender, education, ethnicity, age, chronic illness, choice of medical institution and hospitalization in the corridor) as compared to not expressing any negative voice.

The estimates in Table 4 show that the likelihood of expressing negative criticism is lower for men than women, and that the odds of voicing negative opinion are considerably higher for Jews and for those with academic education than for non-Jews and for those without academic education, respectively. This is consistent with the analysis of satisfaction as a quantitative measure which we showed earlier. The figures are also consistent and confirm the arguments advanced in the literature that women, educated people and members of socially advantaged groups are more likely to express complaints and to voice criticism regarding their care experience.

**Table 4:** Logit regression for predicting odds for expression of (negative) voice

Variables	Expressing voice
*silence= 0; voice= 1*	
*Constant*	−0.223
*Gender (male=1)*	−0.224**
*Education (academic= 1)*	0.294**
*Ethnicity (Jew=1)*	0.610**
*Age*	−0.010**
*Chronic (chronic=1)*	0.226**
*Accommodation (room=1)*	−0.247**
*Hospital choice (choice= 1)*	−0.129**
Log likelihood	14170.96
Cox & Snell R-square	0.028
Nagelkereke R-square	0.039
*N= 11,198*	

### 3.2 Analyzing satisfaction and expression of criticism jointly

In light of the findings that reveal very high levels of satisfaction with the hospitalization and medical experience, on one hand, and the wide expression of criticism, on the other hand, we question what the high satisfaction scores actually mean. To address this issue, we examine the relationship between verbal expression of criticism regarding the hospitalization experience (open ended question) and the satisfaction measures (close ended question). We do so, by estimating logit equation (for each of the three indicators of satisfaction) in which the verbal expression of criticism is introduced to the set of predictors of satisfaction. The results of the analysis are presented in Table 2, model 2. Linear regression predicting satisfaction on 5-point scale produces highly similar results.

As might be expected, the findings listed in Table 2, model 2, show that there is a strong correlation between voicing negative opinion and patient satisfaction and negative criticism is likely to decrease satisfaction. The net impact of ‘negative voice’ on satisfaction is also demonstrated by the unique increment and contribution of ‘complaints’ to the explained variance of satisfaction. An increment to variance in each linear equation (not presented) by the voice variables was almost doubled. It is also noteworthy that unsatisfactory experiences tend to have a crossover effect where a negative experience in one domain appears to reduce satisfaction with regard to all domains of treatment. That is, criticism and complaints regarding the medical care provided by either nurses or doctors is associated not only with lower satisfaction with nurses and physicians but also with lower satisfaction with the environmental context, and vice versa.

An additional question, one that seeks to unpack the meaning of patient satisfaction, is whether people that voiced verbal criticism also gave high satisfaction scores and do they differ in this respect from persons who made no or positive comments. Such finding would support the argument that high quantitative scores are not sufficient to capture the hospitalization experience. We address this question in Table 5. The figures in the table show the distribution of satisfaction scores in the three areas we have been discussing, conditioned on whether the respondent made a verbal comment and if so whether the comment was treatment or hospital conditions related. What we find, as might be expected, is that the percentage of patients giving high scores (4, and especially 5) in each of the three areas, is higher among those who had no verbal criticism. Yet, a substantial proportion of patients who were critical of some aspect of the treatment or hospitalization conditions also gave high satisfaction scores (4 or 5). Over half of those who expressed complaints concerning the treatment of doctors or nurses had scores of 4 or 5 on the satisfaction scale, with over one-fifth giving the top satisfaction score of 5. The pattern with respect to hospitalization conditions shows a somewhat better fit between voiced complaints and satisfaction scores, where only 5 percent of those making a verbal complaint regarding hospitalization conditions had a satisfaction score of 5, compared to 20 percent among those who made no verbal comment.

**Table 5:** The distribution of satisfaction scores related to hospital experience, doctor and nurses treatment, by whether (or not) respondent provided verbal criticism and the issue of verbal criticism

	Hospital conditions			Doctors			Nurses		
Satisfaction Score	Verbal Voice			Verbal Voice			Verbal Voice		
	No comment	Treatment related	Hospital conditions	No comment	Treatment related	Hospital conditions	No comment	Treatment related	Hospital conditions
*1.00–1.99*	0.8	3.3	3.5	0.6	5.6	1.1	0.4	2.9	0.5
*1.00–2.99*	4.4	12.1	16.6	3.0	11.9	4.1	1.8	8.6	3.2
*3.00–3.99*	26.9	36.5	42.0	12.2	24.7	15.6	12.6	25.2	16.4
*4.00–4.99*	48.0	39.3	32.9	38.4	34.0	40.1	45.2	42.0	47.4
*5.0*	19.9	8.8	5.0	45.9	23.8	38.9	40.0	21.3	32.5

	100.0	100.0	100.0	100.0	100.0	100.0	100.0	100.0	100.0

N	5431	1560	1535	6582	1908	1886	6464	1849	1850

In sum, although expression of verbal criticism is associated to some degree with level of satisfaction, we find a strong tendency for patients to report high satisfaction scores even when they had certain negative experiences which they choose to voice. One might argue that the problems the verbal comment refers to might be too minor to affect overall satisfaction. We cannot refute this with the data at hand. Yet, we do point out that expressing voice does require extra effort on the part of the patient; thus, the concern would have to be sufficiently strong. We further note that the tendency to give high satisfaction scores even when voicing a complaint is much more common with respect to doctors and nurses than hospitalization conditions, suggesting that patients may have greater reservations about giving low scores to medical staff, resulting in upwardly biased satisfaction scores.

## 4 Discussion

This study interrogated the meaning of satisfaction with the hospitalization experience based on a large-scale national survey conducted in Israel. Consistent with previous research, the findings of the present research indicate that patients' satisfaction (measured on likert-type scales) is very high and that the variance around the measures of satisfaction is low. Yet, what these high scores mean is not readily apparent. In contrast to the closed-ended questions, verbal responses to an open-ended question regarding the hospitalization experience reveal widespread criticism of the medical experience regarding a variety of issues.

It is possible of course that a respondent could be highly satisfied in general and still provide criticism of a particular aspect of the hospitalization experience. In this regard, we note that the formulation of the open-ended question in the Israeli hospital survey seems to be more encouraging of negative comments than positive comments; also asking for suggestions for improvements. Nonetheless, our analyses indicate that negative verbal comments were associated with lower satisfaction scores on average and both the claims and the tone of verbal responses suggest lower satisfaction with the hospital experience than would be surmised from the quantitative scores alone. It is possible that patients are reluctant to give low and negative evaluation of the medical care in general, and it is possible that at the time of answering the questionnaire, the intensity of the experience was blurred or they are given with empathy to the deteriorating state of the public health system. Therefore, one central conclusion of this research suggests that scores of satisfaction obtained through standard questionnaires are not sufficiently sensitive and do not capture quality of medical care, especially in view of positively skewed scores and the small variation of the quantitative measures. The very fact that the explanatory power of the satisfaction model is substantially enhanced when the coded verbal information is added speaks to this point. This being said, it should be noted that there is still much to explore regarding the relationship and tension between quantitative measures and responses to open-ended questions. This could be addressed in the future by means of in-depth de-briefing interviews with a subset of respondents when a further round of the same survey is fielded.

Overall, the study highlighted the fact that responses to the open-ended question provide a deeper and more nuanced understanding of the hospitalization experience and serve as an important source of information that can contribute to improving the health care system. Yet, major drawback to including open-ended questions to satisfaction surveys is the demand it places on respondents and time and cost involved in analyzing such data. As to respondents, the large number of persons who opted to add verbal responses indicates that the opportunity to make a point in one’s own words is, in fact, welcome by most. Advances in natural language processing are now more readily available reducing the costs associated with coding and analyzing such data and render more sophisticated surveys more feasible. Taking a more comprehensive approach to measuring satisfaction that provide the opportunity for 'voice' is likely to improve the understanding of the strengths and weaknesses of the public health system and promote the legislator's goal - narrowing gaps and achieving equality in the health system.

### 4.1 Avenues for further research

From the data at hand, it is not possible to fully understand the relationship and tensions between the responses to the open-ended and closed-ended questions for some respondents. One way in which this understanding could be advanced in the future would be to add to a round of the same survey in-depth debriefing interviews among a subset of respondents.

## Funding

None.

## Ethical approval

Not required.

## Declaration of Competing Interest

The authors declare that they have no known competing financial interests or personal relationships that could have appeared to influence the work reported in this paper.

## Footnotes

1 Although the Ministry of Health conducted patient satisfaction surveys in hospitals, in 2016 and 2018 as well, only in the 2014 survey was verbal information collected in addition to the closed-ended questions. Therefore, this dataset affords a unique opportunity to study the relationship between the two.
